# Norovirus evolves as one or more distinct clonal populations in immunocompromised hosts

**DOI:** 10.1128/mbio.02177-23

**Published:** 2023-10-31

**Authors:** Natthawan Chaimongkol, Nathânia Dábilla, Kentaro Tohma, Yuki Matsushima, Allison Behrle Yardley, Eric A. Levenson, Jordan A. Johnson, Courtney Ahorrio, Andrew J. Oler, Daniel Y. Kim, Menira Souza, Stanislav V. Sosnovtsev, Gabriel I. Parra, Kim Y. Green

**Affiliations:** 1 Caliciviruses Section, Laboratory of Infectious Diseases, National Institute of Allergy and Infectious Diseases, National Institutes of Health, Bethesda, Maryland, USA; 2 Laboratory of Virology and Cell Culture, Institute of Tropical Pathology and Public Health, Federal University of Goiás, Goiânia, Goiás, Brazil; 3 Division of Viral Products, Food and Drug Administration, Silver Spring, Maryland, USA; 4 Bioinformatics and Computational Biosciences Branch, Office of Cyber Infrastructure and Computational Biology, National Institute of Allergy and Infectious Diseases, National Institutes of Health, Bethesda, Maryland, USA; Duke University School of Medicine, Durham, North Carolina, USA

**Keywords:** norovirus, chronic infection, immunocompromised, RNA populations

## Abstract

Noroviruses are a major cause of acute gastroenteritis worldwide and can establish chronic infection in immunocompromised individuals. To investigate the mechanisms of norovirus evolution during chronic infection, we selected seven representative patients from a National Institutes of Health study cohort who sustained norovirus infection for periods ranging from 73 to 1,492 days. Six patients shed viruses belonging to a single genotype (GII.2[PNA], GII.4 New Orleans[P4], GII.4 Den Haag[P4], GII.3[P21], GII.6[P7], or GII.14[P7]) over the period examined, while one patient sequentially shed two genotypes (GII.6[P7] followed by GII.4 Sydney[P31]). Norovirus genomes from consecutive stool samples were sequenced at high resolution (>3,300 reads/nucleotide position) using the Illumina platform and subjected to bioinformatics analysis. Norovirus sequences could be resolved into one or more discrete clonal RNA genomes that persisted within these patients over time. Phylogenetic analyses inferred that clonal populations originated from a single founder virus and not by reinfection with community strains. Estimated evolutionary rates of clonal populations during persistent infection were similar to those of noroviruses from acute infection in the global database, suggesting that inherently higher RNA-dependent polymerase error rates were not associated with the ability to persist. The high-resolution analysis of norovirus diversity and evolution at the population level described here should allow a better understanding of adaptive mutations sustained during chronic infection.

**IMPORTANCE:**

Noroviruses are an important cause of chronic diarrhea in patients with compromised immune systems. Presently, there are no effective therapies to clear the virus, which can persist for years in the intestinal tract. The goal of our study was to develop a better understanding of the norovirus strains that are associated with these long-term infections. With the remarkable diversity of norovirus strains detected in the immunocompromised patient cohort we studied, it appears that most, if not all, noroviruses circulating in nature may have the capacity to establish a chronic infection when a person is unable to mount an effective immune response. Our work is the most comprehensive genetic data set generated to date in which near full-length genomes from noroviruses associated with chronic infection were analyzed by high-resolution next-generation sequencing. Analysis of this data set led to our discovery that certain patients in our cohort were shedding noroviruses that could be subdivided into distinct haplotypes or populations of viruses that were co-evolving independently. The ability to track haplotypes of noroviruses during chronic infection will allow us to fine-tune our understanding of how the virus adapts and maintains itself in the human host, and how selective pressures such as antiviral drugs can affect these distinct populations.

## INTRODUCTION

Noroviruses are an important cause of acute gastroenteritis that occurs in all age groups (1). The virus is highly infectious and transmitted easily by person-to-person contact or exposure to a common source. The acute symptomatic phase of the illness characteristically lasts from 24 to 48 hours, with projectile vomiting and diarrhea as common clinical features (2). As symptoms resolve, viral RNA may be detected in the stool for a median of 4 weeks (3), and viral clearance has been linked to the development of an active adaptive immune response (4). However, some immunocompromised patients are unable to clear an acute norovirus infection, leading to chronic infection with prolonged periods of viral shedding (5). The overall prevalence of norovirus infection in immunocompromised patients enrolled in clinical protocols at the National Institutes of Health (NIH) Clinical Research Center in Bethesda, MD, was approximately 13%, with evidence for chronic infections in nearly 44% of the patients testing positive for norovirus (6). Symptoms varied in duration and severity over time with diarrhea as the most common clinical symptom. Targeted antiviral therapy for noroviruses is not yet available but is needed (7, 8).

Noroviruses belong to the family *Caliciviridae* and have a positive-sense, single-stranded RNA genome of approximately 7.5 kb in length that is organized into three open reading frames (ORFs). The ORF1 encodes a polyprotein that is post-translationally cleaved by the viral proteinase into six non-structural (NS) proteins (NS1/2, NS3, NS4, NS5^VPg^, NS6^Pro^, and NS7^Pol^) involved in viral replication. The ORF2 and ORF3 encode the major (VP1) and minor (VP2) capsid proteins, respectively (9). The major capsid protein VP1 is composed of a conserved shell (S) domain and a surface-exposed protruding (P) domain, the latter of which is organized into the P1 and P2 subdomains (10). The noroviruses are currently classified into at least 10 genogroups (G), based on differences found in the major capsid protein (VP1) encoded in ORF2, and each genogroup can be further divided into several VP1-based genotypes (11). To address genetic diversity in ORF1, a genotyping system was proposed for the RNA-dependent RNA polymerase (12). To date, more than 40 capsid and 60 polymerase genotypes have been recognized (11), and this dual genotyping system allows the detection and tracking of recombinant noroviruses. Among circulating noroviruses, GII.4 VP1 variants paired with one of several different polymerase genotypes have been the major cause of gastroenteritis outbreaks worldwide (13–15). Previous studies have described the evolutionary pattern of GII.4 as “evolving” because of the continuous accumulation of mutations in the antigenic sites of the VP1 P domain that could lead to the emergence of new variants of GII.4 (16–18). In contrast, non-GII.4 viruses may undergo less intra-genotypic diversification over time. The adaptive and evolving evolutionary pattern of GII.4 may explain, in part, its predominance over other genotypes.

While research on noroviruses has intensively focused on understanding evolutionary patterns and mechanisms, several aspects, including determinants of viral transmission and knowledge of reservoirs for the virus, remain inconclusive. Immunocompromised individuals with chronic norovirus infections have been considered as a possible reservoir for the emergence of new antigenic variants, but no predominant epidemic viruses have yet been directly linked to this source (19–22). The purpose of this study was to evaluate the genetic diversity of noroviruses over time during chronic infection to gain insight into their evolutionary patterns and molecular epidemiology. This knowledge could guide the design and evaluation of therapeutic approaches for the treatment of chronic norovirus disease.

## MATERIALS AND METHODS

### Patient cohort

The seven patients in this study (designated Patients 10, 11, 29, 37, 38, 53, and 129) were enrolled in NIH Institutional Review Board (IRB)-approved clinical research protocols at the NIH Clinical Center, and patient care included management of a primary or secondary immunodeficiency (Table S1). These patients were selected based on the availability of sequential norovirus-positive stool samples that would allow the analysis of several different norovirus genotypes. Upon detection of norovirus RNA in stool by the NIH Department of Laboratory Medicine (DLM), patients were invited to participate also in an IRB-approved viral clinical samples protocol in the Laboratory of Infectious Diseases. Following informed consent, norovirus-positive stool samples were retrieved from the DLM and labeled according to the patient number and order of collection over time (e.g., NIH10.1 is the first stool sample collected from Patient 10).

### Viral RNA extraction, genotyping, and high-throughput sequencing

Stools specimens were prepared as 10% (wt/vol) suspensions in the phosphate-buffered saline, and a 50-µL aliquot was used to extract RNA with the MagMax Viral RNA Isolation Kit (ThermoFisher Scientific, CA, USA). One-step real-time reverse transcription-quantitative polymerase chain reaction (RT-qPCR) was performed to determine norovirus genome copy numbers per gram stool using the TaqMan Fast Virus 1-Step Master Mix (ThermoFisher Scientific). The viral titers were determined using a standard curve generated from seven consecutive 10-fold dilutions of RNA control, ranging from 10^9^ to 10^3^ copies/mL. Subsequently, the viral copy number per gram stool was calculated, considering the elution volume of extracted RNA and the dilution of the stool suspension. To identify the norovirus VP1 (G.genotype classification number) and polymerase [P] genotypes, PCR products were generated in a one-step RT-PCR reaction that spanned the junction between ORF1 and ORF2 and corresponded to partial polymerase (NS7) and capsid (VP1) sequences (23). The PCR products were then purified using the Qiagen Gel Extraction Kit (Qiagen, CA, USA) before DNA sequencing (6). The genotypes were assigned using a web-based norovirus typing tool (24). A subset of samples from each patient was selected for analysis of the full-length norovirus genome by high-throughput sequencing (HTS) as previously described (16). Briefly, primer Tx30SXN (GACTAGTTCTAGATCGCGAGCGGCCGCCCTTTTTTTTTTTTTTTTTTTTTTTTTTTTTT) was used to initiate cDNA synthesis from the viral RNA with the Maxima H Minus First Strand cDNA Synthesis Kit (ThermoFisher Scientific). The cDNA served as a template to generate a genomic-length PCR amplicon of approximately 7.6 kb with 5′-end primer GII1-35 (GII1-35: GTGAATGAAGATGGCGTCTAACGACGCTTCCGCTG) and 3′-end primer Tx30SXN using SequalPrep Long PCR Kit (ThermoFisher Scientific). All amplicon products were resolved in 1% agarose gels, and the resulting full-length viral genome amplicons were excised and purified using the Qiagen Gel Extraction Kit (Qiagen). The recovered amplicons were quantified using the Qubit dsDNA HS Assay Kit (ThermoFisher Scientific) and subjected to HTS library preparation. The library for next-generation sequencing (NGS) was prepared using the Nextera DNA Flex Library Prep Kit (Illumina, CA, USA), and the paired-end 2 × 250 bp sequence reads were obtained using the MiSeq system (Illumina). Reads for each patient sample were quality-filtered (base quality score ≥20) and mapped against a reference genome of the corresponding genotype to construct a genomic consensus sequence as described by Tohma et al. (25) in the HIVE bioinformatics platform (26).

### Norovirus genomic assembly and clonal population analysis

The genomic consensus sequence of an early sample was used as a reference to align the reads from subsequent sequential samples (Table S1). Read alignments and the reference sequence for each sample analyzed were then submitted to the Sequence Profiling tool in the HIVE platform to retrieve output information, including consensus sequences, coverage, and single-nucleotide polymorphism (SNP) frequencies (Fig. S1A). The number of clonal populations present in each sample was defined using Population Analysis as implemented in the HIVE-Hexahedron Coordinated Clonal Analysis tool with a 10% threshold for bifurcation call, considering the error rates associated with the enrichment (RT-PCR full length) and NGS methods described previously (22). Clonal populations predicted by the tool were visualized with a Sankey diagram, as illustrated by the appearance of virus samples in this study with either one (NIH29.1) or two (NIH37.34) major clonal RNA populations (Fig. S1A). As a control to test the ability of the HIVE tool to detect multiple clones, a mixing experiment was performed (Fig. S1B). Briefly, chemically synthesized sequences corresponding to the genomes of GII.4 variant MD145 (GenBank no. AY032605) (27) or Rockville (GenBank no. KY424328), sharing 89.3% nucleotide identity, were engineered into the pCMV6-Entry plasmid downstream of a T7 RNA polymerase promoter. Full-length RNA molecules were transcribed *in vitro* to serve as templates in RT-PCR reactions for the generation of full-length amplicons representing each GII.4 variant. The gel-purified amplicons were mixed at defined ratios and subjected to Illumina sequencing. A representative analysis of the NGS data in HIVE showed the presence of two distinct clonal populations in the Sankey diagram (Fig. S1B). Post-processing filters (sequence size: ≥500 bp, coverage: >50×, and clone support: ≥5 SNPs) were applied to all samples to remove short contigs with low coverage as previously described (28). The full-length and partial norovirus sequences of major clones were subsequently retrieved and assembled for further comparative analyses.

### Phylogenetic and evolutionary analyses

To determine the genetic relationship and clustering pattern of noroviruses associated with chronic infection, clonal population data sets derived from each patient’s samples were aligned using ClustalW and Muscle algorithms. The multi-sequence alignments were tested according to the best nucleotide substitution model following the Bayesian information criterion as implemented in MEGA X (29). The maximum-likelihood (ML) phylogenetic trees based on the nucleotide sequences of each ORF were constructed. Within each ORF tree, sub-clusters consisting of at least four sequences and having at least one of the clonal sequences from the first sample of each patient were reconstructed for further analyses.

To determine the divergence pattern and clock-likeness signal of the noroviruses in this study, the ML phylogenetic trees of each ORF and sub-cluster were used to perform root-to-tip regression analysis using TempEst v1.5.3 software (30). The best-fitting root option was applied selectively to the data set for minimizing the sum of the squared residuals from the regression line to improve the temporal signal. Next, we performed molecular clock analysis to investigate the evolutionary history and to estimate the evolutionary rate of norovirus within immunocompromised hosts as implemented in BEAST v2.6.3 (31). The alignment data set with at least 10 nucleotide sequences of each ORF was used as input under a coalescent constant population prior to the strict clock model. Bayesian Markov Chain Monte Carlo (MCMC) chain was run from 5 to 100 million generations with subsampling every 500–10,000 iterations under the best-fit substitution models corresponding to each data set. The first 10% of logs from MCMC runs was discarded as burn-in before the trees were summarized. All effective sample size values were higher than 200, which was shown to confirm convergence using Tracer v1.7 (32).

To characterize the genetic relationship between norovirus sequences in this study with those of the same genotype in the global data set, we created five separate data sets of full-length VP1 sequences for GII.2, GII.3, GII.4, GII.6, and GII.14 genotypes retrieved from the GenBank database. These large sequence data sets were generated according to a previous report (22) with slight modification by excluding highly similar strains that were collected in an identical year in the same study site/country. Within each genotype, multiple alignments of VP1 nucleotide sequences were initially prepared for MEGA X (29) software and were further subjected to phylogenetic analysis using the ML method under best-fit substitution models with 100 replicates of the bootstrap analysis. Phylogenetic trees inferred in this study were visualized using FigTree v1.4.4 (available at http://tree.bio.ed.ac.uk/software/figtree/).

### Amino acid sequences analysis

Amino acid substitutions acquired over time were determined by analysis of ORFs in the sub-clusters identified from the phylogenetic analysis described above. Within each analysis, the amino acid sequence (major viral clone/major viral haplotype) of the first sample from each patient was used as the reference sequence to compare with those of sequential samples. The percent amino acid difference in each ORF was calculated and plotted against the length of time examined. To assess correlation between amino acid variation and time, the *R*-squared value (*R*^2^) was determined by the linear regression analysis.

To measure the amino acid sequence variability of the VP1 capsid protein within immunocompromised patients over time in comparison with those of VP1 in the global norovirus data set, two sets of aligned amino acid sequences, including the global (background) and patient (query) sequences, were created according to genotype or variant and subjected to Shannon entropy-two analysis (https://www.hiv.lanl.gov/content/sequence/ENTROPY/entropy.html). A consensus amino acid sequence was initially generated and aligned against all sequences within each data set from a patient. The Shannon entropy value of each amino acid site was measured with higher entropy values reflecting greater amino acid variation.

In addition to the major structural protein VP1, amino acid sequences of the minor structural protein VP2 were analyzed. Amino acid sequences from the first and last collected samples from each patient and reference sequences were aligned to compare variation in or near the predicted ORF1 proteolytic cleavage sites. The aligned sequences were visualized and edited with MEGA X software. Amino acid sequences from the global references or all sequential samples from the patients were also aligned to examine variation within the non-structural proteins. Pairwise distances to each amino acid position were analyzed by SSE software (33). Residues in or near the active sites of protease (NS6^Pro^) and RNA-dependent RNA polymerase (NS7^Pol^) were compared by WebLogo server (34).

## RESULTS

### Norovirus genotypes associated with chronic infection

**Fig 1:**
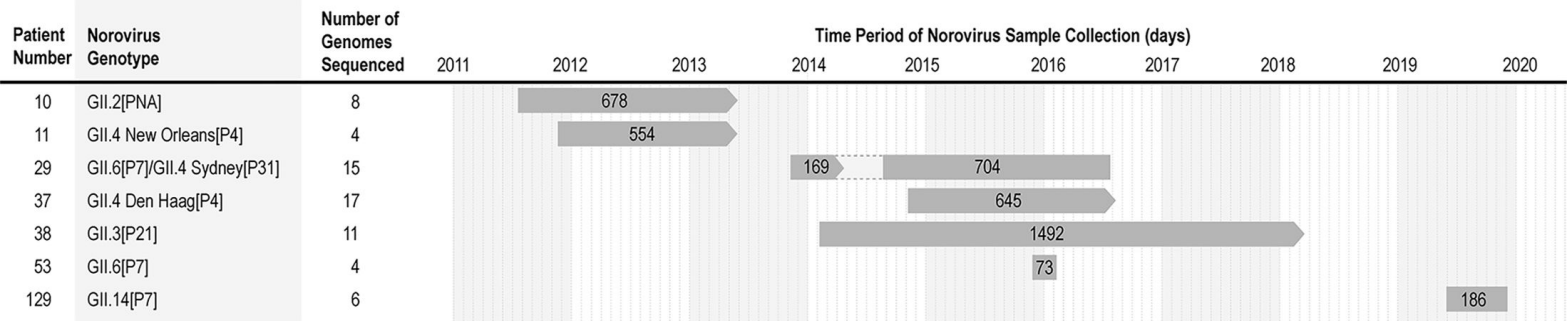
Immunocompromised patients, infecting norovirus genotypes, and period of sample collection.

Seven patients at the NIH Clinical Research Center who were enrolled in protocols that managed a primary or secondary immunodeficiency and who were diagnosed with norovirus infection were selected for this viral genomics study (Fig. 1; Table S1). All patients shed a single norovirus genotype during the follow-up period, with one exception. Patient 29 (immunosuppressed due to stem cell transplantation for the treatment of leukemia) sequentially shed two different genotypes: GII.6[P7] followed by GII.4 Sydney[P31], with evidence for a period of co-infection with the two viruses until GII.4 Sydney[P31] emerged as the sole genotype detected (Table S1). Several different genogroup II (GII) genotypes of VP1 and polymerase were detected in this population (Fig. 1), consistent with the epidemiological predominance of GII noroviruses (14, 35). Among the viruses analyzed, the polymerase gene of the GII.2 norovirus in Patient 10 could not be genotyped with the norovirus typing tool and was designated as “not assigned” (NA). The full-length sequence of this gene shared the highest homology (90%) with a GII.2[P40] norovirus reported from Japan in 2004 (accession number: DQ366347), and this sequence served as the reference to guide the reads mapping and genome assembly of the NIH10 samples. Immune globulin (IG) therapy administered orally or by the intravenous (IV) route to 6 of the 7 patients in this cohort did not effectively reduce viral genome copies quantified from sequential samples collected during the patient’s visits (Table S1). The eventual clearance of norovirus in the two patients with primary immunodeficiency (Patients 10 and 37) correlated with the gradual reconstitution of a functional immune system following gene therapy. Patients 29, 53, and 129 sustained norovirus infection until death.

### Norovirus evolves as one or more distinct clonal populations in immunocompromised hosts

**Fig 2:**
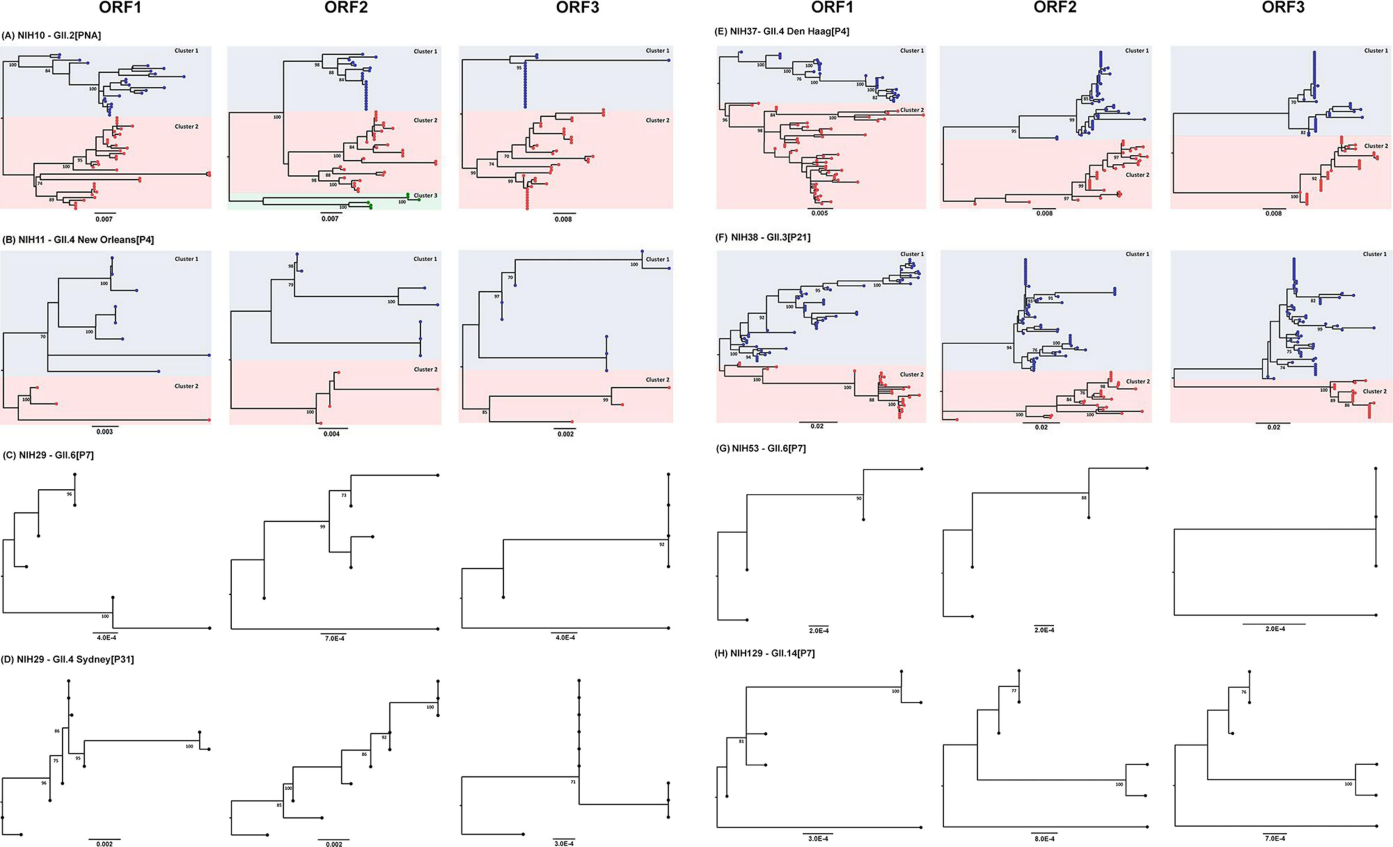
Intra-host diversity of noroviruses within each patient. Nucleotide sequences representing each viral population identified in sequential samples from each patient by the HIVE-Hexahedron were retrieved for phylogenetic analysis. Phylogenetic trees were constructed based on the nucleotide sequences’ alignments of each ORF of the NIH10 viruses from Patient 10 (A), the NIH11 viruses from Patient 11 (B), the NIH29 viruses from Patient 29 (C and D), the NIH37 viruses from Patient 37 (E), the NIH38 viruses from Patient 38 (F), the NIH53 viruses from Patient 53 (G), and the NIH129 viruses from Patient 129 (H) using the maximum-likelihood method with 100 bootstrap replicates, as implemented in MEGA X software. The viruses from Patients 10 (A), 11 (B), 37 (E), and 38 (F) contained multiple populations represented in dendrograms as clusters 1, 2, or 3 and marked with blue, red, and green-filled circles, respectively. Sequences from viruses shed by Patients 29 (C and D), 53 (G), and 129 (H) each contained one major population represented in the dendrogram as one evolving cluster marked with black-filled circles.

Next-generation sequencing of the norovirus genome in each stool specimen showed a high level of SNP frequencies across the genome. We applied the HIVE-Hexahedron Coordinated Clonal Analysis tool to analyze the NGS data and found that specimens with the highest frequency of SNPs could be resolved into multiple discrete clones (haplotypes) of RNA genomes (Table 1; Fig. S2). Phylogenetic reconstruction of the near full-length genomic sequences from consecutive samples confirmed that noroviruses in the immunocompromised patients in this study persisted as one or more clonal RNA genomes within the host over the time period followed (Fig. 2; Fig. S3A through C). The intra-host RNA populations in Patients 10, 11, 37, and 38 appeared to evolve independently because the phylogenetic root-to-tip divergence plots presented higher clock-like signals when they were analyzed separately as phylogenetic sub-clusters compared to the overall sequences (Fig. S4).

**TABLE 1:** Estimated year of norovirus infection for the immunocompromised patients in this study by TMRCA^*b*^ analysis of VP1

Patient number	Norovirus genotype	No. of sequential samples analyzed	Year of 1st collection	Period of collection (days)	ORF2 TMRCA in days [95% HPD^*b*^ interval]	Estimated year of infection	No. of sub-population genomes over time^*a*^
10	GII.2[PNA]	8	2011	678	2,298 [1,519, 3,212]	2005	2
11	GII.4 New Orleans[P4]	4	2011	554	746 [350, 1,204]	2009	2
29	GII.6[P7]	6	2013	169	8 [1, 31]	2013	1
	GII.4 Sydney[P31]	9	2014	704	488 [456, 507]	2013	1
37	GII.4 Den Haag[P4]	17	2014	645	893 [646, 1,166]	2012	2
38	GII.3[P21]	11	2014	1,492	1,947 [1,486, 2,461]	2009	2
53	GII.6[P7]	4	2015	73	25 [1, 54]	2015	1
129	GII.14[P7]	6	2019	186	12 [1, 41]	2019	1

^*a*^ Number of sub-population genomes refers to number of clusters identified by phylogenetic analysis. In one patient (Patient 10), evidence was seen for an additional third cluster in ORF2 (Fig. 2).

^*b*^ HPD, highest posterior density; TMRCA, time to the most recent common ancestor.

### Single founder virus rather than multiple primary infections

**Fig 3:**
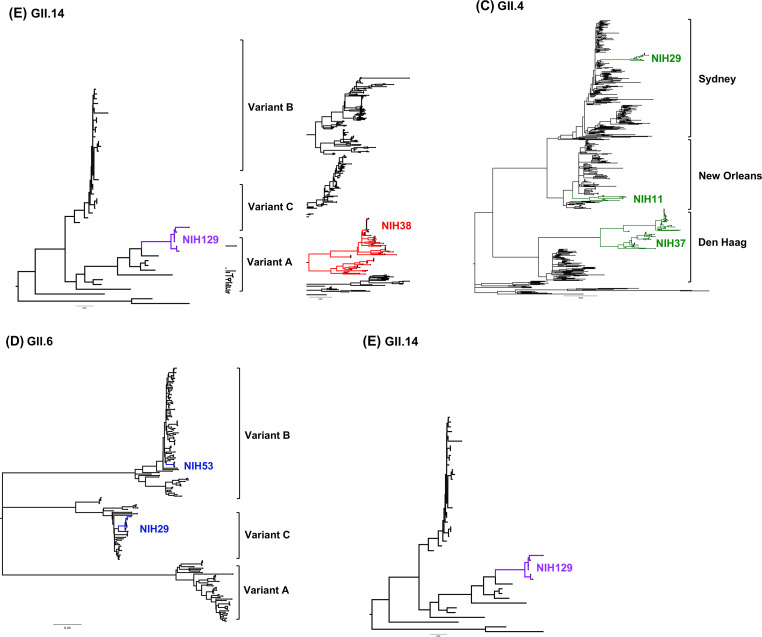
Comparison of noroviruses from immunocompromised patients with those in the global database. Phylogenetic trees based on the alignments of full-length VP1(ORF2) sequences of noroviruses were inferred using the maximum-likelihood method with 100 bootstrap replicates, MEGA X software. Nucleotide sequences of 398 of GII.2 (A), 305 of GII.3 (B), 531 of GII.4 (C), 173 of GII.6 (D), and 46 of GII.14 (E) were included in this analysis. Sequences identified from immunocompromised patients in our cohort study were clustered separately from sequences collected globally. Variant designations for the GII.6 and GII.4 norovirus genotypes are indicated to the right of the dendrogram.

We constructed phylogenetic trees to compare the ORF2 sequences of different clonal populations within each patient to those circulating globally (Fig. 3). Phylogenetic analysis showed that norovirus sequences from each patient formed a distinct and closely related cluster, suggesting that viral populations within patients originated from a single founder virus and not from multiple new infections. In addition, the correlation between time to the most recent common ancestor (TMRCA) of the ORF2 and global scale epidemiology allowed for timing estimates of exposure and infection (Table 1). Patient 10 was chronically infected with GII.2[PNA] norovirus. The VP1 sequences from this patient formed a distinct cluster (with a high bootstrap value of 88) from other sequences of a rarely detected GII.2[P40] genotype (36) that was reported from 2001 to 2004 in the global data set (37) (Fig. 3A). The predicted ORF2 TMRCA of the virus shed by Patient 10 in 2011 was 2,298 days [95% highest posterior density (HPD) interval, 1,519–3,212]. The mean estimate of the TMRCA suggests that norovirus from this patient diverged from a common ancestor around 2005. In Patient 38, stool samples tested positive for GII.3[P21] norovirus from 2014 to 2018. This recombinant virus has been detected globally since 2000 (38) and has continued to circulate in young children as recently as 2016–2020 (35). The estimated TMRCA of NIH38 sequences was calculated to be 1,947 days (95% HPD interval, 1,468–2,461) which dates back to 2009. When considered at the TMRCA inferred for NIH10 and NIH38 sequences, the timepoints were consistent with exposure of Patients 10 and 38 to the viruses during the years when it circulated, suggesting evidence of chronic infection for as long as 6 and 5 years before enrolling in our cohort study, respectively.

Likewise, the correlation between ORF2 TMRCA and global scale epidemiology was applied to the GII.4 variants detected in this study. Patient 37 was first diagnosed with GII.4 Den Haag virus infection in November 2014. Globally, the Den Haag variant emerged in 2006 and was replaced by the GII.4 New Orleans variant in 2009, although it continued to be detected at low levels until 2015 (13). The TMRCA for the tree root of Patient 37 was estimated to be 893 days (95% HPD interval, 646–1,166) which dates back to approximately 2012. This timepoint suggests that the patient may have sustained chronic infection for as long as 2 years during a co-circulation of old and newly emerging GII.4 variants. Patient 11 tested positive for the GII.4 New Orleans variant in 2011. The estimated TMRCA inferred for NIH11 sequences dates back to 746 days (95% HPD interval, 350–1,204) in 2009. This finding again suggests that Patient 11 was first exposed to the GII.4 New Orleans variant when the virus was detected across the globe during 2010–2011. Patient 29 tested positive for the GII.4 Sydney variant in 2014. The estimated TMRCA was 488 days (95% HPD interval, 456–507), which would correspond to 2013, a year when the GII.4 Sydney variant was predominant (14).

This study detected GII.6 noroviruses in Patients 29 and 53 and GII.14 in Patient 129 in combination with the GII.P7 polymerase genotype. However, due to the short period of sample collection, the estimated TMRCAs of these patient sequences dated back closely to the beginning of sample collection (Table 1).

### Evolutionary rates of norovirus in immunocompromised hosts

**Fig 4:**
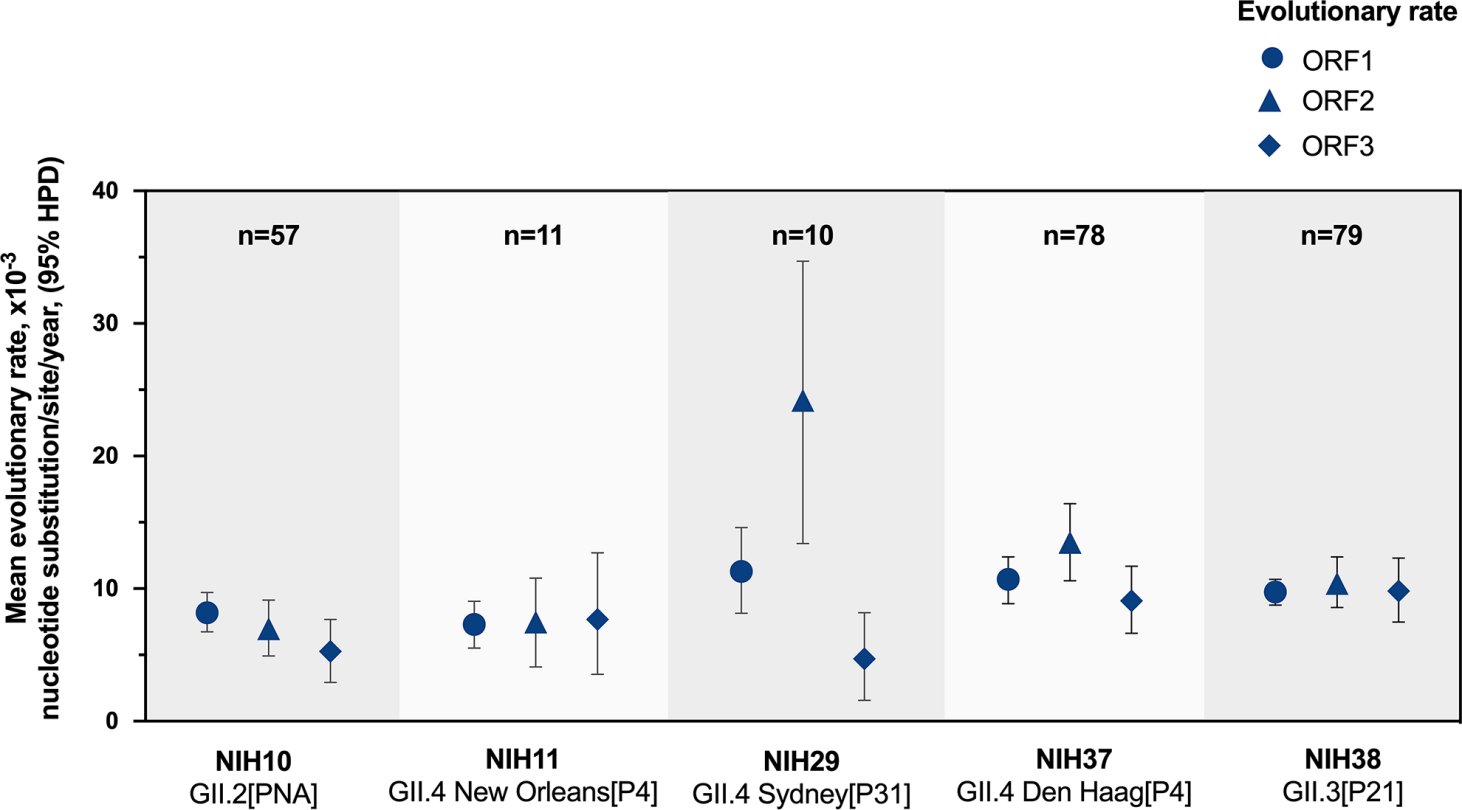
Within-host evolutionary rates of noroviruses in immunocompromised patients shedding for ≥1 year. Mean evolutionary rates (nucleotide substitution/site/year × 10^−3^) were shown together with their 95% HPD intervals of each ORF.

We estimated the evolutionary rates of noroviruses in the patient cohort using BEAST v2.6.3 with strict clock model to assume substitution rates across phylogenetic branches. The coalescent constant population prior was selected for the rate of evolution. The lengths of time for sample collection allowed an analysis based on substitutions per site per year. Evolutionary rates were estimated from the clonal sequences retrieved from each patient, considering each ORF of the genome separately (Fig. 4). The rates ranged from 7.30 × 10^−3^ to 11.30 × 10^−3^ in ORF1, 6.97 × 10^−3^ to 24.20 × 10^−3^ in ORF2, and 4.71 × 10^−3^ to 9.82 × 10^−3^ in ORF3. Most published reports have noted the high frequency of nucleotide (SNPs or SNVs) or amino acid substitutions as factors to discuss the evolutionary trends of norovirus in these patients (20, 39–41). Our data indicate that unusually high SNP frequencies in certain NGS-derived norovirus consensus sequences are likely due to the presence of distinct clonal populations (haplotypes), and not because of inherently higher error rates of the infecting virus.

### Amino acid sequence analysis

**Fig 5:**
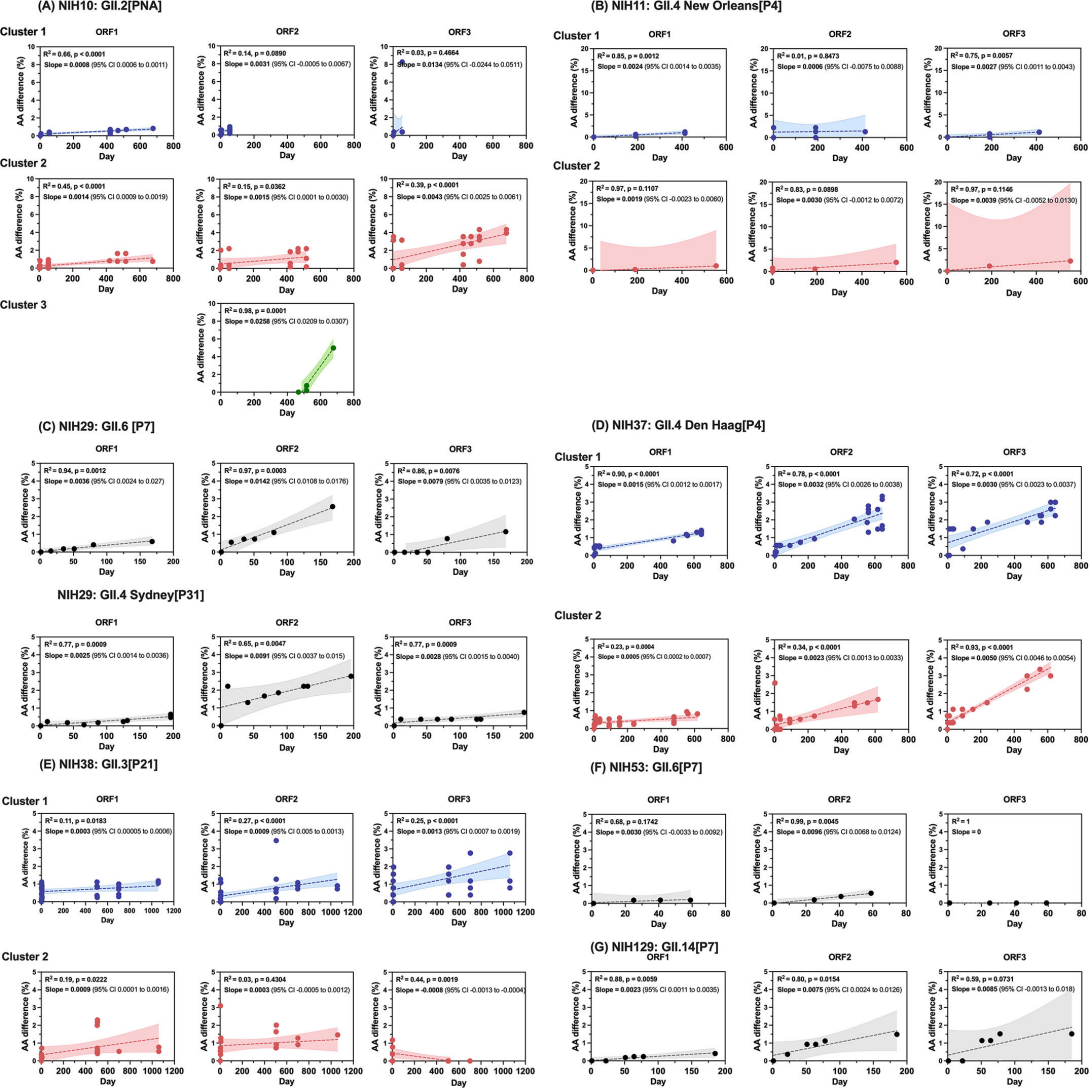
Distribution of amino acid sequence variability in GII.4 and non-GII.4 noroviruses during chronic infection. The percentage of amino acid difference in each ORF was calculated per specimen relative to the sequence of the first sequential sample within the same cluster (population). Scatterplots demonstrate that the overall amino acid sequences of each ORF varied by patient. The relationship between sequence differences and shedding periods is shown by correlation coefficient, *R*^2^ and slope values. The viruses from Patients 10 (A), 11 (B), 37 (D), and 38 (E) contained multiple populations represented in dendrograms as clusters 1, 2, or 3 and marked with blue, red, and green-filled circles, respectively. Sequences from viruses shed by Patients 29 (C), 53 (F), and 129 (G) each contained one major population represented in the dendrogram as one evolving cluster marked with black-filled circles.

This study further investigated the impact of genetic diversity within a host on amino acid changes over time. First, we examined the major capsid protein, VP1, and found that several substitutions were positively correlated with the duration of sample collection times within individuals (Fig. 5). This relationship is clearly observed among GII.4 noroviruses, including those of Patients 29 (*R*^2^ = 0.65 in the linear regression model) and 37 (cluster 1; *R*^2^ = 0.78 and cluster 2; *R*^2^ = 0.34). Our finding is consistent with a previous study (22) that certain codons encoded in ORF2 are evolving under strong selective pressure.

Shannon entropy analysis showed a significantly higher amino acid variation located on the P2 domain of VP1 capsid protein compared to shell and P1 domains, where most positions have an entropy level below the cutoff value (<0.25) (Fig. S5A and B). Accordingly, the accumulation of VP1 amino acid variations within each host over time was analyzed (Tables S2 and S3). Among GII.4 noroviruses, amino acid variation at several previously mapped antigenic sites was observed (Fig. S5C; Table S2). Notably, some substitutions in this study were rarely observed in the data set of globally circulating viruses but became the predominant residue at certain positions. These included Glu at position 372 (site A), Gly at position 407 (site E), and Leu at position 352 (site G) in the GII.4 New Orleans virus from Patient 11, as well as Gln at position 297 (site A), Asp at 377 (site C), and Pro at 395 and Lys at 397 (site D) in the GII.4 Sydney virus from Patient 29. In addition, Asp at 376 and Pro at 377 (site C), and Asn at 255 (site I) were observed in the GII.4 Den Haag VP1 from Patient 37 (Table S2). It is interesting to note the following: (i) as previously noted for GII.4 noroviruses (42, 43), two individuals (Patients 11 and 37) shed GII.4 viruses that showed convergence in residues previously shown to co-evolve and (ii) one ORF2 clone in Patient 11 contained an amino acid deletion at residue 395 that was thought to be displaced globally when an insertion emerged at this site in the Farmington Hills variant during 2002 and 2003 (44). The presence of substitutions in the proposed antigenic sites of VP1 suggests the development of phenotypic differences over time, as shown previously (45). Because both VP1 and the minor structural protein VP2 are present in the viral capsid and interact, we compared the complete VP2 amino acid sequences of viruses collected over time in each patient. The viruses from Patients 10 (GII.2[PNA]) and 37 (GII.4 Den Haag[P4]) sustained several substitutions through the entire VP2 protein, while few changes were observed in those from Patient 29 (GII.6[P7] and GII.4 Sydney[P31]). We focused on the proposed VP1-interacting domain (corresponding to nucleotide positions 300–600) within the VP2 gene. This domain, along with the P2 domain of VP1, was previously defined as one of two local hypervariable regions among norovirus GII.4 strains US 95/96, Farmington Hills, Hunter, Sakai, 2006a, and 2006b (46). In our analysis, even though non-synonymous substitutions were occasionally observed, this VP2 region did not appear to be hypervariable within and among the sequentially collected viruses in this study. However, we found a recurring nucleotide swap between cytosine and uracil at position 435 of the VP1-interaction domain in the Den Haag variant (2006) identified in Patient 37. This synonymous substitution was reported also in a previous study of an immunocompromised patient who chronically shed a GII.4 2006b norovirus (46).

We next reviewed the genetic diversity of the non-structural protein sequences encoded in ORF1 (Fig. S6 and S7). Comparison of the ORF1 dipeptide proteolytic cleavage sites (P1/P1′), known to be highly conserved within a norovirus genotype, showed that these sites remained highly conserved over long periods of chronic infection. However, variation in the surrounding sequences occurred in some genomes. The NIH10 (GII.PNA) virus contained Thr or Ala residues distinct from reference strains in the P3 and P4 residues of clusters 1 and 2 surrounding the NS4^p22^/NS5^VPg^ cleavage site and the last sequential sample in cluster 1 of the strain had a Leu in the P4 position of NS5^VPg^/NS6^Pro^. The NIH29 (GII.P7) virus contained two unique substitutions: a Thr residue in the P2′ position of the NS3^NTPase^/NS4^p22^ cleavage site and a Ser in the P4′ position of the NS6^Pro^/NS7^Pol^ site. The last sequential sample of NIH53 (GII.P7) also included a Ser in the P2′ position of the NS6^Pro^/NS7^Pol^ site. The NIH11 (GII.P4) had a specific Ala in the P2 position of the NS4^p22^/NS5^VPg^ and the NIH37 (GII.P4) viruses showed amino acid changes between clusters 1 and 2 in the P3′ position of the NS3^NTPase^/NS4^p22^ site. The NIH38 (GII.P21) included a Leu in the P4′ position of the NS3^NTPase^/NS4^p22^ site and changed P3 residue of NS5^VPg^/NS6^Pro^ from a Ser to an Asn in cluster 1 (Fig. S6). These results suggest that substitutions can occur in positions close to the cleavage sites during intra-host evolution, although their effect on viral replication is currently unknown. An overall comparison of ORF1 sequences showed that viruses from chronic infection in immunocompromised patients sustained similar pairwise distances on amino acid variations throughout the ORF1 region, with no discernable hot spots of variation (Fig. S7). This differs from viruses in the global database that are mostly associated with epidemic disease, where hotspots of variability with high pairwise distances have been observed in the NS1/2^N-term^ and NS4^p22^ proteins except for GII.P21 (22). Moreover, no specific patterns of acquired amino acid substitutions in the ORF1 NS proteins could be observed in this study as infection persisted over time. Residues in or near the active sites of NS6^Pro^ and NS7^Pol^ were conserved except for one variation between Ile and Thr at amino acid 135 on the S1 pocket, which interacts with P1 residues of the substrate, in the NS6^Pro^ of NIH10 (GII.PNA) (Fig. S7).

## DISCUSSION

Noroviruses are recognized as an important cause of chronic diarrhea in immunocompromised individuals, but the mechanisms by which they establish and maintain persistent infection are poorly understood. The ability of RNA viruses to persist is influenced by both viral and host factors (47). For noroviruses, the failure to eliminate the virus by a competent immune response is a known host factor that plays a role in viral persistence (2, 48). The purpose of this study was to define the genetic features of noroviruses associated with persistent infection as a first step toward understanding the contribution of virus factors to this complex interaction.

Noroviruses from several different genotypes exhibited complex patterns of genomic diversity over time in our patients, with viruses evolving as one or more distinct clonal RNA populations, or haplotypes. Our findings are consistent with those of Doerflinger et al. (49), Venturini et al. (50), and Ruis et al. (51). who reported evidence for distinct haplotypes within the population of certain GII.4 noroviruses during chronic infection. Our analysis showed that additional genotypes (GII.2 and GII.3) have the capacity to evolve as distinct haplotypes during chronic infection, suggesting that the generation of distinct RNA populations within a host may be a shared feature among noroviruses.

Once individual populations were identified, phylogenetic analysis of the noroviruses in this study showed that each patient maintained chronic infection with haplotypes that were distinct from the global database of norovirus sequences. Our results are consistent with the unique clustering of norovirus strains from immunocompromised patients reported in other studies (6, 40, 52, 53). Although immunocompromised hosts have not been identified as major reservoirs for the emergence of new strains (21, 49, 54), the possibility exists as noroviruses from immunocompromised patients have been associated with person-to-person transmission (19, 55) and infectivity in an enteroid cell culture system (56). Continuing monitoring and characterization of norovirus strains associated with both epidemic disease and chronic infection will be needed to reconstruct transmission events and define potential genetic bottlenecks (19, 41, 55, 57, 58).

The diversity of genotypes observed among the noroviruses in our study is consistent with the wide range of genotypes that have been linked to persistent infection (6, 39, 59, 60). Our time clock analysis of the multiple defined haplotypes in our norovirus collection showed that the rates of ORFs 1, 2, and 3 for both GII.4 and non-GII.4 noroviruses were only slightly higher compared to those reported in previous studies of inter-host evolution during acute infections (16, 17, 22), or in immunocompetent children with up to 98 days of virus shedding (54). This finding indicates that the marked diversity of a human norovirus genome during chronic infection is driven primarily by prolonged, continuous replication with an error-prone RdRp that functions with a fidelity similar to that of an acute infection. We used the TMRCA inferred from available intra-host norovirus sequences to predict the time of infection of single founder viruses. It was noteworthy that the TMRCA estimates for the noroviruses in this study were consistent with the molecular epidemiology and temporal occurrence of these viruses in the community. Although the precise timing of the primary infection is often unknown for immunocompromised individuals with chronic infection, the TMRCA analysis may lead to a better understanding of the duration of the infection and whether greater variation over time is a factor in treatment.

Viral population analysis has identified distinct haplotypes in other RNA viruses associated with long-term infection such as HIV (61, 62) and hepatitis C virus (63, 64). These multiple populations, apparently arising from a number of selective pressures such as host response or antiviral drugs, are thought to increase the capacity of these viruses to persist within the host. Investigations are underway to understand how individual haplotypes behave and interact during chronic infection and transmission. Notably, defective genomes have been identified in some long-term viral infections that may have lower transmission rates or cause attenuated disease (65). The characteristics of distinct viral populations during long-term norovirus infection are not known. One explanation for the origin of norovirus haplotypes within a host could be an original exposure event that allows infection with multiple founder viruses, such as the exosome model of transmission that has been proposed for certain RNA viruses (66). In this way, multiple and even distinct viruses could be transmitted to a new host in a single event. Alternatively, a single founder virus may have given rise to multiple haplotypes over time due to prolonged and unabated replication. Patients with single clonal norovirus populations (Patients 29 with first GII.6[P7] infection, 53, and 129) were followed for shorter periods (73–186 days) compared to those (Patients 10, 11, 29 with second GII.4[P31] infection, 38, and 56) with multiple populations (554–1,492 days), suggesting that longer infection times may indeed contribute to diversification. Moreover, it is possible that replication in different target cells or tissues in the host could exert varying selective pressures. The selective pressures in immunocompromised patients that influence norovirus diversification may vary also in terms of the underlying degree and nature of the immune impairment (primary versus secondary) or drug treatment protocols. For example, the monthly administration of IVIG is often standard care for individuals with severe combined immunodeficiency, and the accumulation of mutations in the epitopes of the norovirus VP1 may result from this treatment as shown here and in other studies (6, 45, 58, 67).

Persistent infection in mice by murine norovirus (MNV) has been linked to certain viral strains (68) and an adaptive mutation at residue 94 in the MNV NS1/2 protein has been identified (69). We did not find evidence of a comparable NS1/2 adaptive mutation in the human noroviruses analyzed in this study, but it should be noted that the patients’ specimens associated with the acute, original infections were not available. Our comparative amino acid analysis confirmed the presence of invariant regions in evolving norovirus populations such as the dipeptide cleavage sites in the ORF1 polyprotein and motifs in the replicative enzymes such as the RdRp expected to be critical for productive infection. The absence of discernable hotspots of variability or conserved acquired substitutions in our analysis does not rule out a role for the non-structural proteins in the establishment of persistence, and expanded genomics analysis of these viruses may be needed to detect patterns. It is also not known whether different clonal RNA populations reflect viable or defective viruses. Efficient reverse genetics and cell culture systems remain technically challenging for the human noroviruses, but such studies will be needed to assess phenotypic differences *in vitro*.

Finally, our findings show the complexity of intra-host diversity of noroviruses during chronic infection but also provide a new strategy for tracking the selective pressures of adaptation and persistence at high resolution. Moreover, the analysis of haplotypes is helpful in the interpretation of NGS data from chronic norovirus infections, which often contain an unusually high number of SNPs across the norovirus genome in these patients (14, 39, 40, 49, 55, 57, 59, 60, 70). Continued high-resolution sequencing at the haplotype level should prove useful in the development and evaluation of therapeutics, including immunotherapy.

## Data Availability

The consensus nucleotide sequence of the norovirus sample used as a reference for alignment of subsequent samples from each patient was submitted to NCBI GenBank and assigned an accession number as follows: OR065073 (NIH10.1), OR051787 (NIH11.1), OR051903 (NIH29.1), OR065090 (NIH29.10), OR051927 (NIH37.1), OR069404 (NIH38.3), OR069405 (NIH53.1), and OR069406 (NIH129.1).

## SUPPLEMENTAL MATERIAL

Figure S1 mbio.02177-23-s0001.pdf HIVE platform pipeline.

Supplemental Figure Legends mbio.02177-23-s0002.docx Legends and references for Fig. S1 to S7.

Figure S2 mbio.02177-23-s0003.pdf Sankey diagrams.

Figure S3 mbio.02177-23-s0004.tif Color coded ML trees.

Figure S4 mbio.02177-23-s0005.pdf RTT analyses.

Figure S5 mbio.02177-23-s0006.tif Entropy analysis of VP1 domains.

Figures S6 and S7 mbio.02177-23-s0007.pdf Nonstructural protein analysis.

Table S1 mbio.02177-23-s0008.xlsx List of patient samples.

Table S2 mbio.02177-23-s0009.xlsx GII.4 patient 11, 29, and 37 VP1 evolution.

Table S3 mbio.02177-23-s0010.xlsx Non GII.4 patient 10, 38, 29, 53, and 129 VP1 evolution.

ASM does not own the copyrights to Supplemental Material that may be linked to, or accessed through, an article. The authors have granted ASM a non-exclusive, world-wide license to publish the Supplemental Material files. Please contact the corresponding author directly for reuse.
