# Supplementary material for: Norovirus evolves as one or more distinct clonal populations in immunocompromised hosts
Source: mBio. 2023 Oct 31;14(6):e02177-23. doi: 10.1128/mbio.02177-23 (PMC10746188; doi:10.1128/mbio.02177-23)
Supplement: Figure S1 — HIVE platform pipeline. [file mbio.02177-23-s0001.pdf]

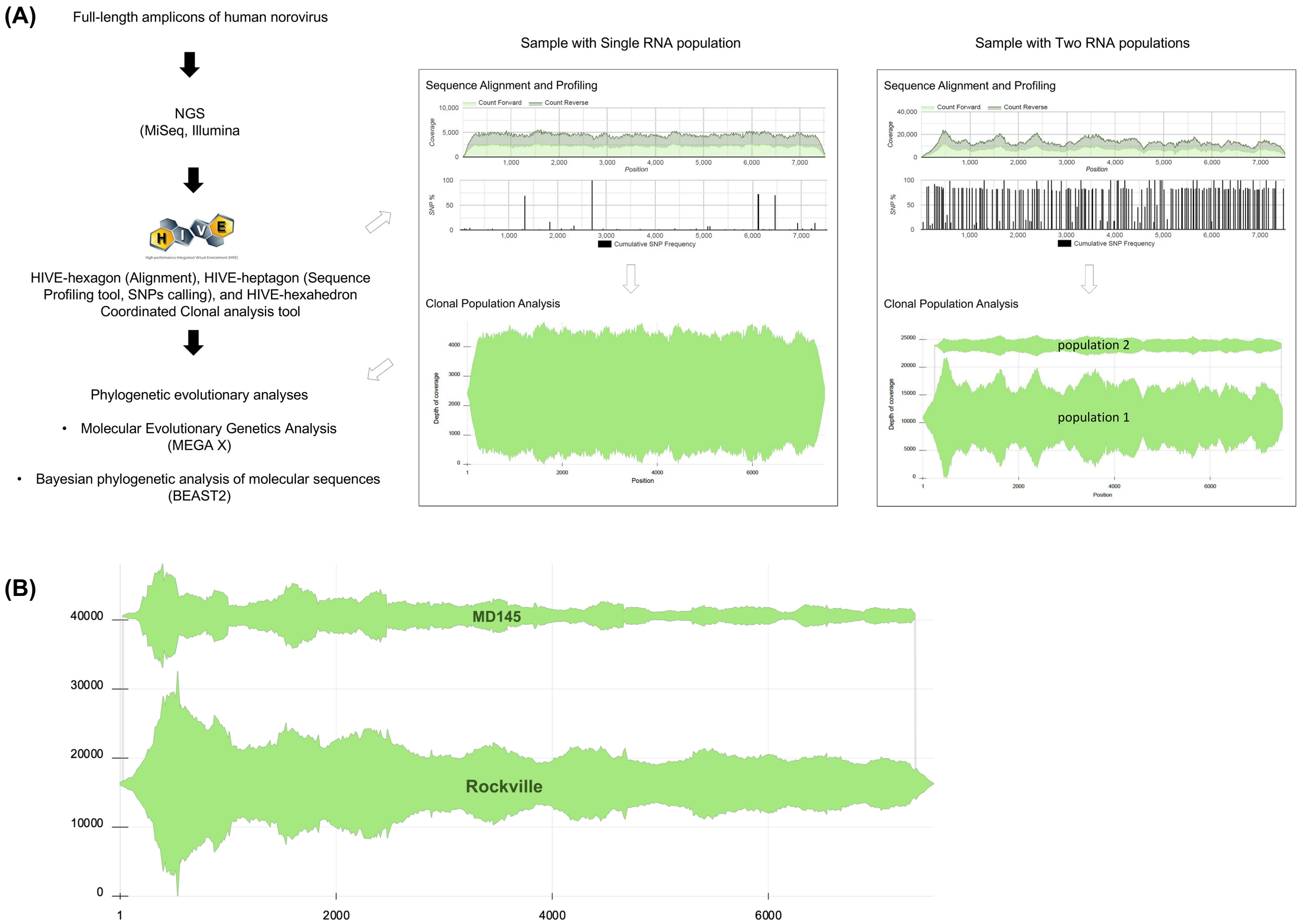
(A)
Full-length amplicons of human norovirus
NGS
(MiSeq, Illumina
High-performance Integrated Virtual Environment (HIVE)
HIVE-hexagon (Alignment), HIVE-heptagon (Sequence Profiling tool, SNPs calling), and HIVE-hexahedron Coordinated Clonal analysis tool
Phylogenetic evolutionary analyses
• Molecular Evolutionary Genetics Analysis (MEGA X)
• Bayesian phylogenetic analysis of molecular sequences (BEAST2)
Sample with Single RNA population
Sequence Alignment and Profiling
Count Forward
Count Reverse
Coverage
Position
SNP %
Cumulative SNP Frequency
Clonal Population Analysis
Depth of coverage
Position
Sample with Two RNA populations
Sequence Alignment and Profiling
Count Forward
Count Reverse
Coverage
Position
SNP %
Cumulative SNP Frequency
Clonal Population Analysis
population 2
population 1
Depth of coverage
Position
(B)
MD145
Rockville
40000
30000
20000
10000
0
1
2000
4000
6000
