# Supplementary material for: Norovirus evolves as one or more distinct clonal populations in immunocompromised hosts
Source: mBio. 2023 Oct 31;14(6):e02177-23. doi: 10.1128/mbio.02177-23 (PMC10746188; doi:10.1128/mbio.02177-23)
Supplement: Supplemental Figure Legends — Legends and references for Fig. S1 to S7. [file mbio.02177-23-s0002.docx]

**SUPPLEMENTAL Figure Legends**

**FIG S1** Flowchart illustrating HIVE algorithms used to identify distinct viral populations in immunocompromised patient infected with norovirus. Full-length norovirus amplicons were amplified and subjected to next-generation sequencing using the Illumina platform. The HIVE algorithms used in this study (HIVE-hexagon, HIVE-heptagon, and HIVE-hexahedron) were applied to sequence alignments, base profiling, SNP variant calling, and viral populations analysis that was visualized by Sankey diagrams. The Sankey diagrams indicate the length of a viral genome spanning from the 5’ -3’ ends along the x-axis and the depth of sequence coverage at each nucleotide along the y-axis. Examples of Sankey diagrams illustrating a single predominant RNA population (left panel, from sample NIH29.1) or two RNA populations (right panel, from sample NIH37.34) are shown. In the case of two RNA populations, distinct populations are defined corresponding to the abundance and length of the contigs namely populations 1 and 2. Detailed information generated in each step can be selectively viewed and downloaded, and nucleotide sequences of assembled genomes were subjected to evolutionary analyses (A). Control experiment illustrating the ability of the HIVE algorithms to distinguish between GII.4 norovirus genomes MD145 (GenBank AY032605) and Rockville (GenBank KY424328) prepared and analyzed as described in Materials and Methods (B).

**FIG S2** RNA population analysis of each norovirus sample as visualized in a Sankey diagram. Sequential norovirus samples from each patient were analyzed in the HIVE-Hexahedron and RNA populations were visualized by Sankey diagrams. In this study, two patterns of RNA populations were observed, either a single predominant RNA population (NIH29, NIH53, and NIH129) or multiple RNA populations (NIH10, NIH11, NIH37, and NIH38)

**FIG S3** Phylogenetic trees constructed from sequences obtained from clonal populations of Patients 10 (GII.2[PNA]), 37 (GII.4 Den Haag[P4], and 38 (GII.3[P21]) by ORFs using the Maximum-likelihood method with 100 bootstrap replicates, as implemented in MEGA X software. The viruses from Patients 10 (A), 37 (B), and 38 (C) were phylogenetically organized into multiple populations, denoted as Clusters 1, 2, or 3 (shown in brackets). Different colors used in the dendrograms represent the respective years of sample collection.

**FIG S4** Root-to-tip (RTT) regression analysis of sequences identified from clonal populations of each patient by ORF. Plots of genetic divergence versus sampling dates are shown for estimated temporal signals in each dataset. In the case of nucleotide sequences grouped into more than one cluster (e.g., sequences identified from Patients 10, 11, 37, and 38), separate datasets were generated and subjected to the analysis. The regression of RTT genetic distance against sampling time points represented higher temporal signals when they were analyzed separately by subclusters compared to the overall sequences.

**FIG S5** Shannon entropy analysis within the major capsid VP1 protein of non-GII.4 and GII.4 noroviruses. Shannon entropy values of each individual codon (represented by a dot) were calculated and plotted for norovirus sequences identified globally (gray dots) and from the immunocompromised patients in this study (blue dots). The blue shaded columns indicate the P2-domain with relatively high variability in amino acid sequences among non-GII.4 (A) and GII.4 (B) noroviruses. Within GII.4 viruses, the distribution of Shannon entropy values appeared to vary across antigenic sites A, C, D, E, G, and I (C) (1-2). The cut-off value is set at 0.25.

**FIG S6** Comparison of amino acid sequences around ORF1 cleavage sites between patient samples and reference strains. Alignments of amino acid P1-P4 sequences around ORF1 cleavage sites with first and last collected samples and reference strains for GII genotypes and other genogroups are shown. Dots represent identity with a GII.P12 reference sequence (GII.P12/MD145-12/GenBank AY032605). Samples are designated by NIH patient number, sequential sample order, clone (c) number as assigned by HIVE and collection day from first sample. Clusters are classified based on phylogenetic analyses in this study.

**FIG S7** Comparison of amino acid sequences between clonal populations from patient samples [NIH10 (GII.PNA) (A), NIH11 (GII.P4) (B), NIH29 (GII.P7) (C), NIH29 (GII.P31) (D), NIH37 (GII.P4)] (E), NIH38 (GII.P21)] (F), and NIH53 and NIH129 (GII.P7)] (G) and (E) in overall ORF1 and around the mapped enzymatic active sites of NS6^Pro^ (3) and NS7^Pol^ (4). Pairwise distance plots to each amino acid position are shown on the left side. The plots are created with parameters including numeric precision of 4, minimum completeness of 50%, length of fragment of 30 and increment between fragments of 3. The x-axis exhibits amino acid positions while the y-axis represents pairwise distances. Residue conservations in or near the active sites of NS6^Pro^ and NS7^Pol^ are shown on the right upper and lower sides, respectively. The x-axis represents amino acid positions whereas the y-axis exhibits the information content of each amino acid position.

**REFERENCES (SUPPLEMENTAL MATERIAL)**

1. Tohma K, Lepore CJ, Gao Y, Ford-Siltz LA, Parra GI. 2019. Population Genomics of GII.4 Noroviruses reveal complex diversification and new antigenic sites involved in the emergence of pandemic strains. *mBio* 10:e02202-19.

2. Tohma K, Ford-Siltz LA, Kendra JA, Parra GI. 2022. Dynamic immunodominance hierarchy of neutralizing antibody responses to evolving GII.4 noroviruses. *Cell Rep* 39:110689.

3. Viskovska MA, Zhao B, Shanker S, Choi JM, Deng L, Song Y, Palzkill T, Hu L, Estes

MK, Venkataram Prasad BV. 2019. GII.4 Norovirus protease shows pH-sensitive proteolysis with a unique Arg-His pairing in the catalytic site. *J Virol* 93:e01479-18.

4. Zamyatkin DF, Parra F, Alonso JM, Harki DA, Peterson BR, Grochulski P, Ng KK. 2008. Structural insights into mechanisms of catalysis and inhibition in Norwalk virus polymerase. *J Biol Chem* 283:7705-7712.
