# Supplementary material for: Norovirus evolves as one or more distinct clonal populations in immunocompromised hosts
Source: mBio. 2023 Oct 31;14(6):e02177-23. doi: 10.1128/mbio.02177-23 (PMC10746188; doi:10.1128/mbio.02177-23)
Supplement: Figure S2 — Sankey diagrams. [file mbio.02177-23-s0003.pdf]

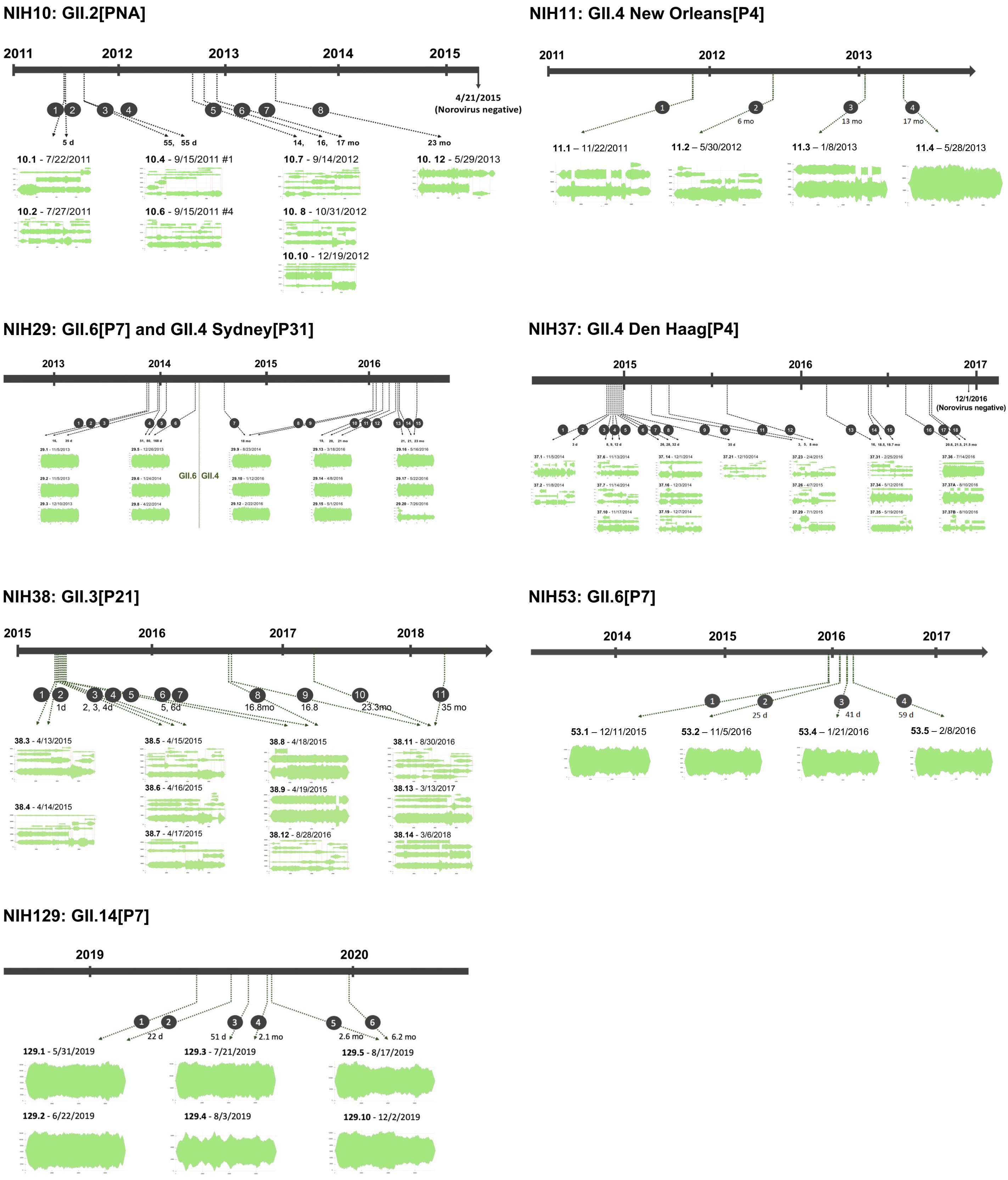

NIH10: GII.2[PNA]
2011
2012
2013
2014
2015
4/21/2015
(Norovirus negative)
5 d
55, 55 d
14, 16, 17 mo
23 mo
10.1 - 7/22/2011
10.4 - 9/15/2011 #1
10.7 - 9/14/2012
10. 12 - 5/29/2013
10.2 - 7/27/2011
10.6 - 9/15/2011 #4
10. 8 - 10/31/2012
10.10 - 12/19/2012
NIH11: GII.4 New Orleans[P4]
2011
2012
2013
6 mo
13 mo
17 mo
11.1 – 11/22/2011
11.2 – 5/30/2012
11.3 – 1/8/2013
11.4 – 5/28/2013
NIH29: GII.6[P7] and GII.4 Sydney[P31]
2013
2014
2015
2016
GII.6
GII.4
NIH37: GII.4 Den Haag[P4]
2015
2016
2017
12/1/2016
(Norovirus negative)
NIH38: GII.3[P21]
2015
2016
2017
2018
1d
2, 3, 4d
5, 6d
16.8mo
16.8
23.3mo
35 mo
38.3 - 4/13/2015
38.5 - 4/15/2015
38.8 - 4/18/2015
38.11 - 8/30/2016
38.6 - 4/16/2015
38.9 - 4/19/2015
38.13 - 3/13/2017
38.4 - 4/14/2015
38.7 - 4/17/2015
38.12 - 8/28/2016
38.14 - 3/6/2018
NIH53: GII.6[P7]
2014
2015
2016
2017
25 d
41 d
59 d
53.1 – 12/11/2015
53.2 – 11/5/2016
53.4 – 1/21/2016
53.5 – 2/8/2016
NIH129: GII.14[P7]
2019
2020
22 d
51 d
2.1 mo
2.6 mo
6.2 mo
129.1 - 5/31/2019
129.3 - 7/21/2019
129.5 - 8/17/2019
129.2 - 6/22/2019
129.4 - 8/3/2019
129.10 - 12/2/2019
