# Supplementary figures and images for: Norovirus evolves as one or more distinct clonal populations in immunocompromised hosts
Source: mBio. 2023 Oct 31;14(6):e02177-23. doi: 10.1128/mbio.02177-23 (PMC10746188; doi:10.1128/mbio.02177-23)

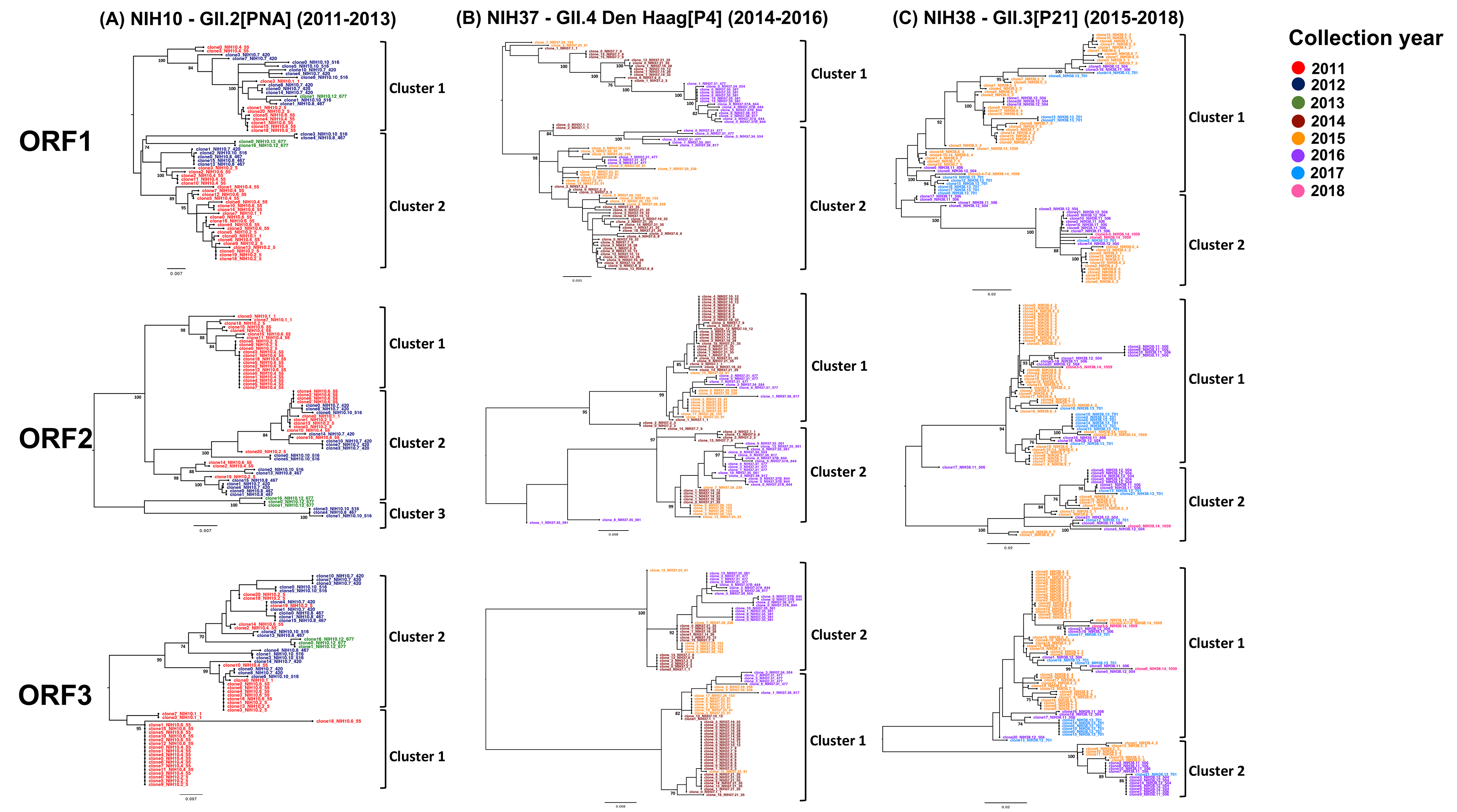

Supplement: Figure S3 — Color coded ML trees. [file mbio.02177-23-s0004.tif]

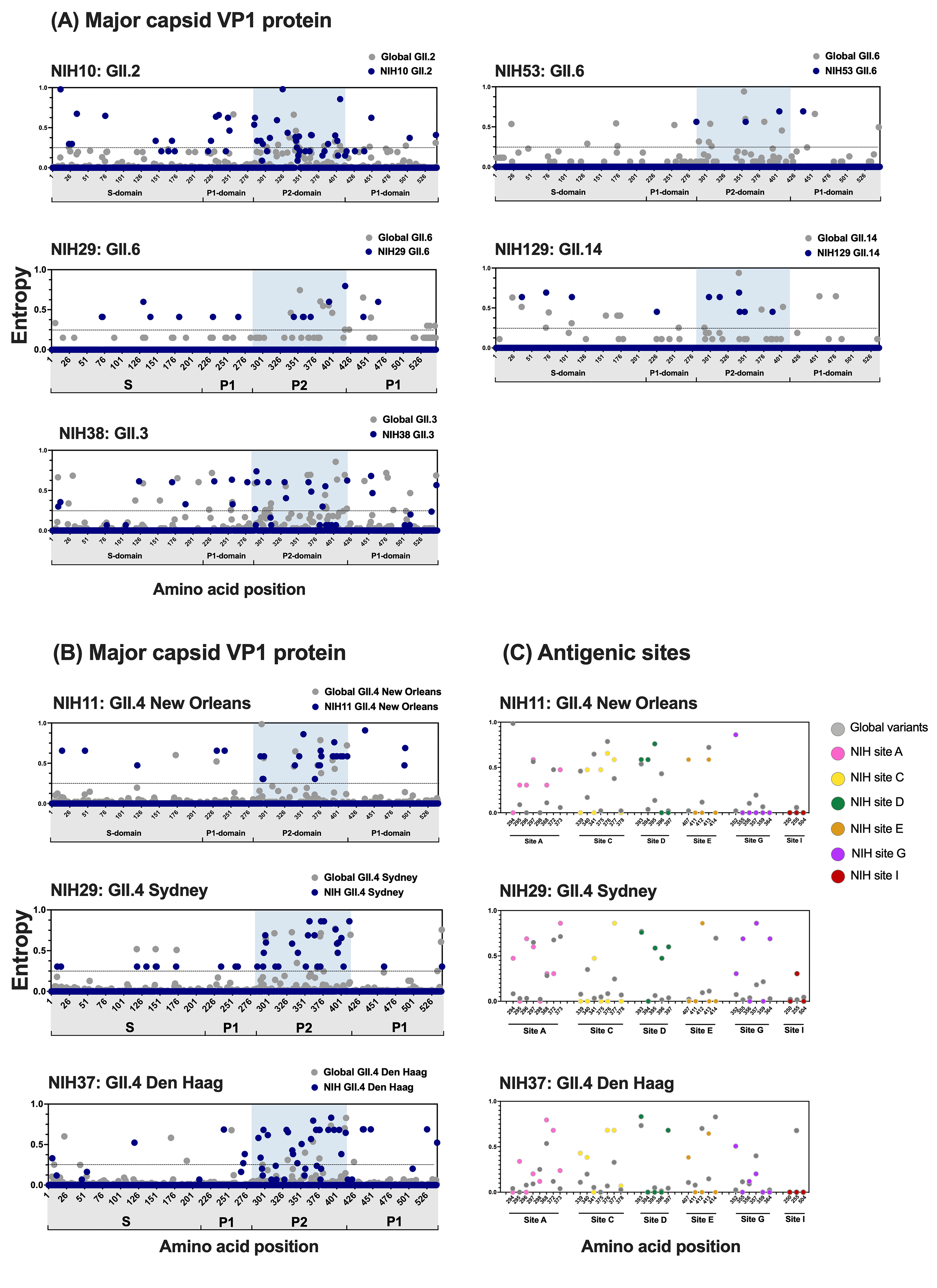

Supplement: Figure S5 — Entropy analysis of VP1 domains. [file mbio.02177-23-s0006.tif]

# Figure S6

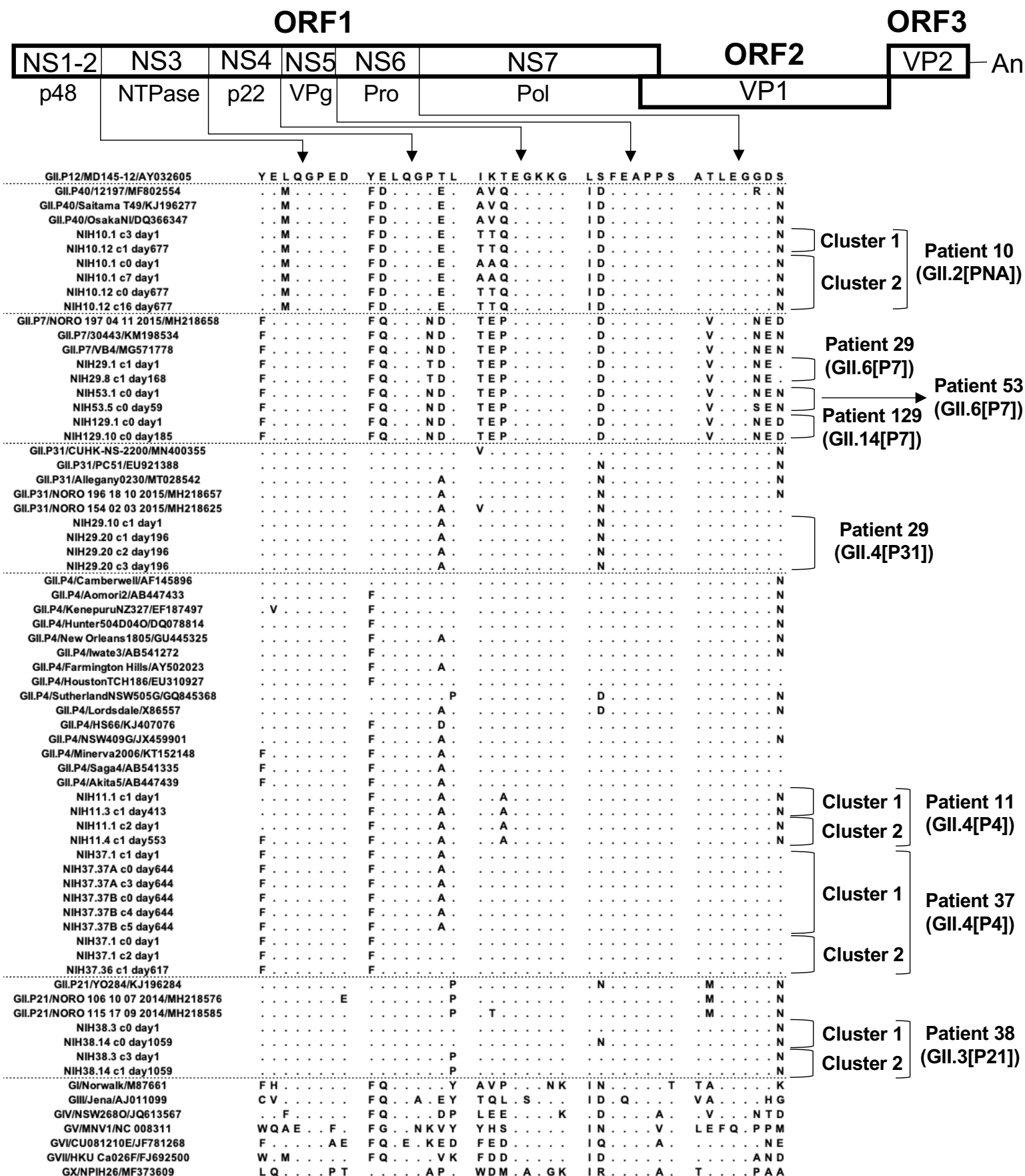

Figure S7

(A) GII.P40

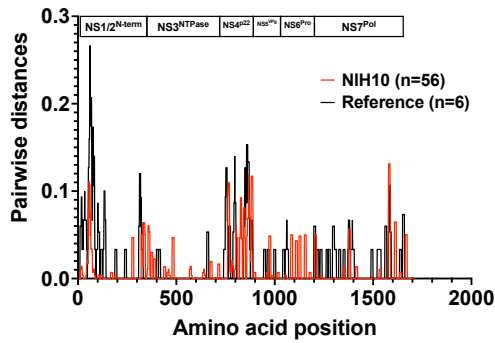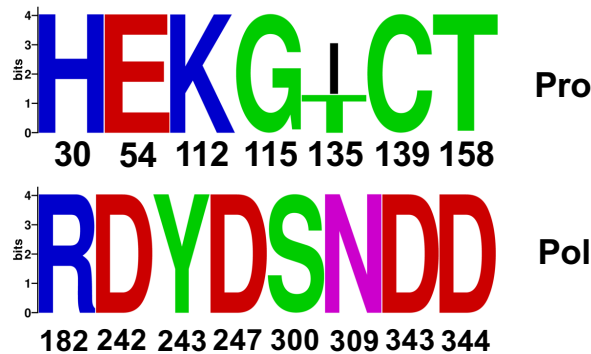

(B) GII.P7

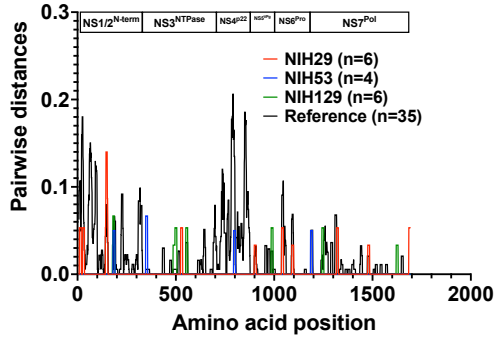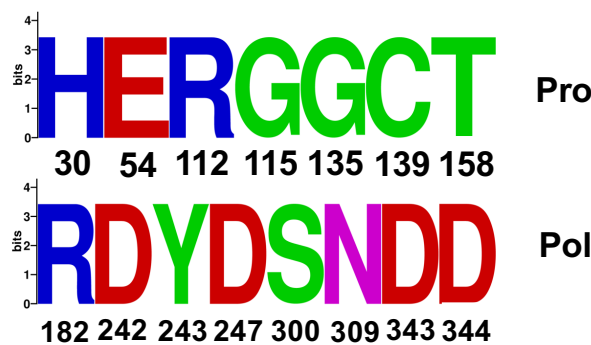

(C) GII.P31

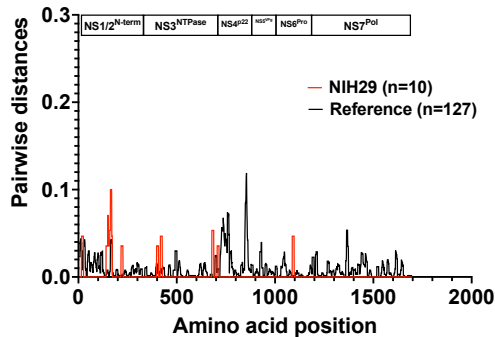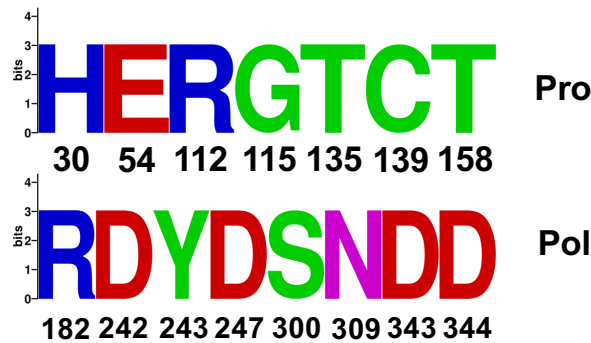

(D) GII.P4

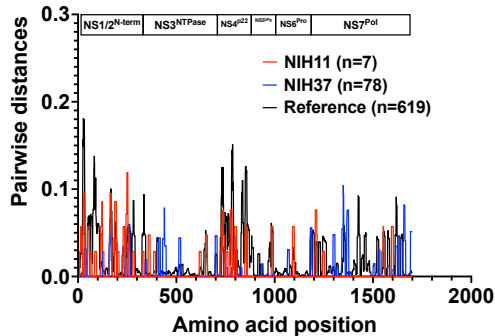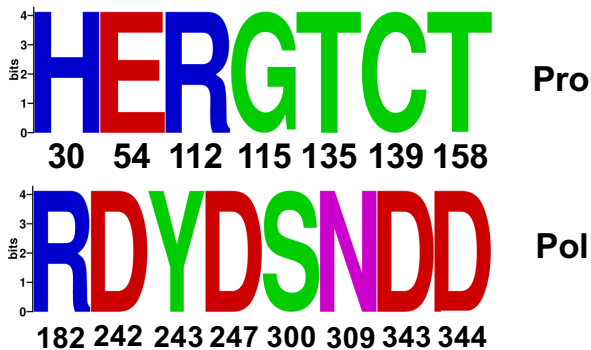

(E) GII.P21

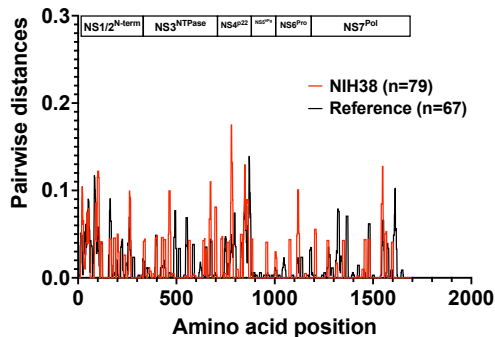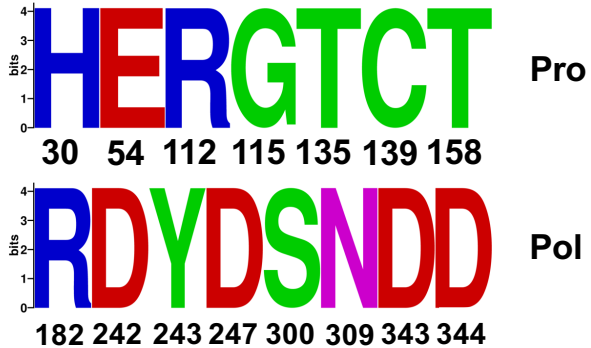

Supplement: Figures S6 and S7 — Nonstructural protein analysis. [file mbio.02177-23-s0007.pdf]
