# Supplementary material for: Norovirus evolves as one or more distinct clonal populations in immunocompromised hosts
Source: mBio. 2023 Oct 31;14(6):e02177-23. doi: 10.1128/mbio.02177-23 (PMC10746188; doi:10.1128/mbio.02177-23)

NIH10: GII.2[PNA]

RTT analysis - overall sequences RTT analysis - by cluster

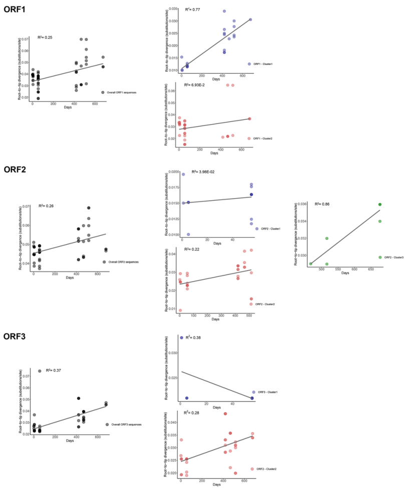

NIH11: GII.4 New Orleans[P4]

RTT analysis - overall sequences RTT analysis - by cluster

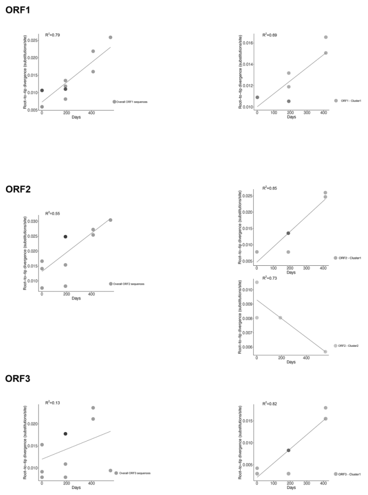

NIH29: GII.6[P7]

RTT analysis

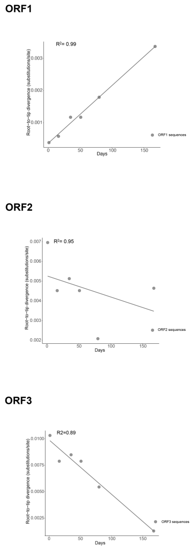

NIH29: GII.4 Sydney[P31]

RTT analysis

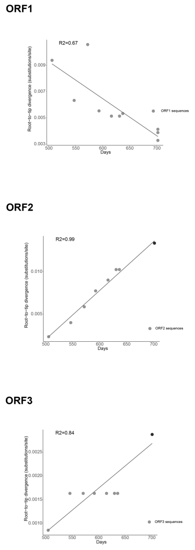

NIH37: GII.4 Den Haag[P4]

RTT analysis – overall sequences

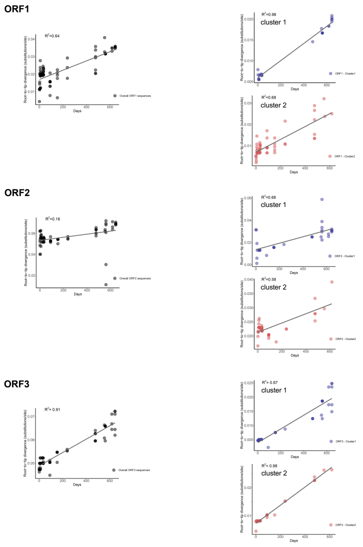

NIH38: GII.3[P21]

RTT analysis – overall sequences

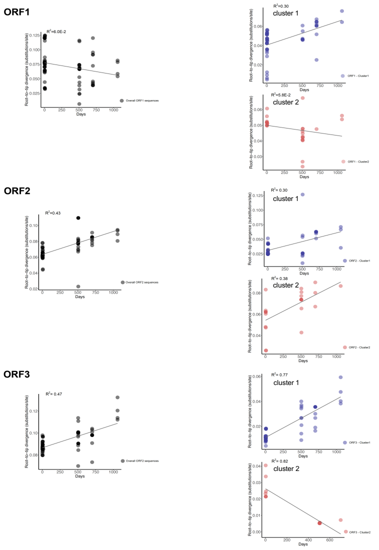

NIH53: GII.6[P7]

RTT analysis

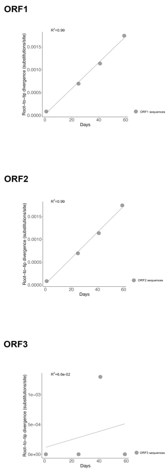

NIH129: GII.14[P7]

RTT analysis

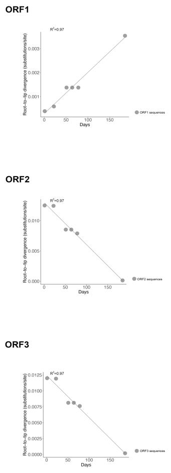

Supplement: Figure S4 — RTT analyses. [file mbio.02177-23-s0005.pdf]
